# The Sanitation of Camps

**Published:** 1917-02-24

**Authors:** Alfred Keogh


### THE SANITATION OF CAMPS.

By SURGEON-GENERAL SIR ALFRED KEOGH, G.C.B.

SPEAKING at a meeting of the Royal Institute of Public Health on the 14th inst., Sir Alfred Keogh said that the sanitation of camps derived its importance not merely because of its significance for armies in the field, but also because the main principles of rural sanitation were bound up therein. He could not help thinking, from all he had heard from people who had been engaged in sanitation in France, Mesopotamia, Salonika, and Egypt, that after the war these problems of rural sanitation would probably derive a new importance from the knowledge which had now been acquired at those various places. There was necessity for a wide diffusion of knowledge on matters of this kind. Referring to the very great decline in the prevalence of enteric fever, he said that in the British Army in France on that date there were five known cases of enteric and eighteen of paratyphoid fever. In addition there were seventy to eighty febrile cases of which the diagnosis had not then been established, so that the figures he had given would probably be increased. Such a number was, of course, negligible. The relatively large amount of paratyphoid at the Dardanelles was doubtless due to the fact that whilst that campaign was being waged no preventive vaccine for the disease was available, while the present very small number of cases was due to this hiatus having been made good. Never again would it be possible for the public or administrators to say that medical science was not applicable to great administrative problems connected with public health. In the distant past the sanitary recommendations made in reference to troops were in the nature of experiments; but all that had now gone, it was replaced by exact knowledge, and the measures were amply justified by the results. One of the most remarkable aspects of the scientific work which had been done during this war was that problems which might have had to wait for years before being investigated had been, under the stress of present conditions, worked at with extraordinary success.

In reference to the prevention of diseases in the Army, he would like to emphasise a point on which he had always felt very strongly. However thoroughly inoculations were carried out, whatever precautions for cleanliness were observed, disease would occur among the troops unless they were well fed. It was necessary to remember that the physiological resistance possessed by the body was the best protection we could have, and he believed much of the disease which existed in armies formerly was due to the absence of a sound system of feeding the men in the field. He considered that some stress ought to be laid on the work which was done some years ago by a committee of experts at the War Office in working out, on a physiological basis, the ration of the soldier. Physiology was too often left out of consideration in these matters; it was pathology all the time. He concluded by paying a high tribute to the work of the R.A.M.C. at the commencement of the war, when three millions of men had to be dealt with, and there were not places for their housing in encampments, so that they had to be billeted in private houses and any place which could be found.

